# Crown Lengthening Procedures: Biological Foundations, Clinical Outcomes, and Contemporary Approaches in Flapless and Flap Techniques—A Narrative Review

**DOI:** 10.3390/dj14070413

**Published:** 2026-07-07

**Authors:** Blagovesta Yaneva, Aleksandra Pecheva, Meri Hristamyan

**Affiliations:** 1 Department of Periodontology and Oral Mucosa Diseases, Faculty of Dental Medicine, Medical University of Plovdiv, 4000 Plovdiv, Bulgaria; 2 Department of Operative Dentistry and Endodontics, Faculty of Dental Medicine, Medical University of Plovdiv, 4000 Plovdiv, Bulgaria; aleksandra.pecheva@mu-plovdiv.bg; 3 Department of Epidemiology and Disaster Medicine, Faculty of Public Health, Medical University of Plovdiv, 4000 Plovdiv, Bulgaria; meri.hristamyan@mu-plovdiv.bg

**Keywords:** crown lengthening, flapless crown lengthening, periodontal surgery, minimally invasive dentistry, biologic width, supracrestal tissue attachment

## Abstract

**Background:** Crown lengthening procedures are widely used in restorative and periodontal therapy to expose sufficient tooth structure for functional and esthetic rehabilitation. Traditionally performed using an open-flap approach, these procedures have evolved with the introduction of minimally invasive flapless techniques. This narrative review aims to summarize the biological foundations, clinical outcomes, and contemporary approaches associated with crown lengthening procedures, with particular emphasis on flapless and flap techniques, healing processes, technological advancements, and clinical decision-making considerations. **Methods:** A narrative literature review was conducted using electronic databases, including PubMed, Scopus, and Web of Science, to identify relevant publications on crown lengthening procedures. The search focused on studies addressing flapless and flap techniques, biological principles, healing dynamics, clinical outcomes, postoperative considerations, and contemporary approaches such as laser-assisted and digitally guided procedures. Titles and abstracts were screened for relevance, followed by full-text assessment of eligible articles. The study selection process was documented using a simplified PRISMA-style flow diagram to enhance transparency. **Results:** The reviewed literature suggests that flapless crown lengthening may be associated with reduced surgical time, less postoperative discomfort, faster healing, and preservation of soft tissue architecture in appropriately selected cases. However, its applicability appears to be influenced by anatomical factors, including bone morphology and the need for adequate visualization during osseous recontouring. Conventional flap techniques continue to be widely used and may offer improved surgical access and visibility, particularly in more complex situations requiring extensive bone modification. Although these procedures have been associated with longer healing periods and greater postoperative morbidity, the heterogeneity of the available evidence and the narrative nature of this review limit definitive comparisons between the two approaches. **Conclusions:** In conclusion, both flapless and flap crown lengthening procedures demonstrate high clinical efficacy when appropriately indicated. The choice of technique should be guided by case-specific factors, clinician expertise, and patient expectations. Further randomized controlled trials are recommended to strengthen the evidence base and establish standardized clinical guidelines.

## 1. Introduction

Clinical crown lengthening is a periodontal surgical procedure aimed at increasing supragingival tooth structure by apical repositioning of the gingival margin, often associated with alveolar bone resection, to facilitate restorative or esthetic dental treatments [1,2,3].

This procedure can be performed using conventional crown lengthening techniques or by innovative minimally invasive and laser approaches. These procedures are indicated when exposure of tooth structure is necessary to improve prosthetic retention, treat caries or subgingival fractures, correct excess visible gingiva, correct abnormal passive eruption, or expose tooth structure to optimize the esthetic results of prosthetic rehabilitation [4,5,6].

Surgical intervention may include gingivectomy, apical repositioned flap, and/or bone resection, with the aim of restoring adequate biological width and providing sufficient tooth structure for the restorative margins while preserving periodontal health [7]. The extent of bone and soft tissue removal is tailored to the dimensions of the supracrestal tissues and the prosthetic needs of each patient. Healing usually involves some degree of coronal soft tissue repair, especially in patients with a thick periodontal biotype, and stabilization of the final gingival margin may take several months.

Surgical crown lengthening uses open-flap and closed-flap techniques to expose sufficient tooth structure while preserving periodontal health. As these approaches vary in their invasiveness, accessibility, and outcomes, comparative evaluation is needed to optimize clinical outcomes [8,9].

The open-flap technique is a conventional method that consists of lifting a full-thickness mucoperiosteal flap, often associated with bone remodeling, to reposition the gingival margin apically. It allows direct visualization for precise bone reduction, but requires sutures and prolonged healing [8,9,10]. The closed-flap technique, also called flapless or minimally invasive, uses ultrasonic piezo surgery or lasers to remove bone without lifting the flap. Guided by probing or imaging, it preserves vascularization, minimizes trauma, and promotes faster soft tissue healing [4,11,12].

A comparison of these techniques reveals that: the open flap provides better predictability in complex cases involving multiple teeth or requiring deep bone resection, while the closed flap reduces pain, keratinized tissue loss, and postoperative morbidity [8,13].

Randomized studies have shown comparable long-term stability of gingival margin position, but closed approaches are preferred for esthetic reasons and patient satisfaction [9].

In addition, modern dentistry favors minimally invasive procedures to limit tissue trauma, improve healing, and improve quality of life, consistent with the evidence supporting flapless methods for isolated teeth in esthetic areas. This approach reduces complications such as gingival recession and preserves the integrity of the biological width [11,12].

Despite the availability of systematic reviews focusing on esthetic crown lengthening and short-term periodontal outcomes, a comprehensive narrative synthesis integrating biological principles, clinical indications, surgical techniques, complications, and emerging technologies remains lacking, thereby justifying the need for the present review.

This narrative review aims to synthesize the available evidence on flapless and flap crown lengthening techniques, focusing on gingival margin stability, periodontal parameters, postoperative morbidity, healing outcomes, esthetic results, and complications, while also addressing biological principles and contemporary adjunctive technologies relevant to clinical decision-making.

A narrative review design was selected due to the broad and multifaceted nature of the topic, which encompasses biological principles, clinical techniques, and emerging technologies in crown lengthening. The available literature is heterogeneous in design and outcomes, making a systematic synthesis inappropriate. Therefore, an integrative narrative approach was used to synthesize and interpret the evidence.

## 2. Materials and Methods

**Figure 1:**
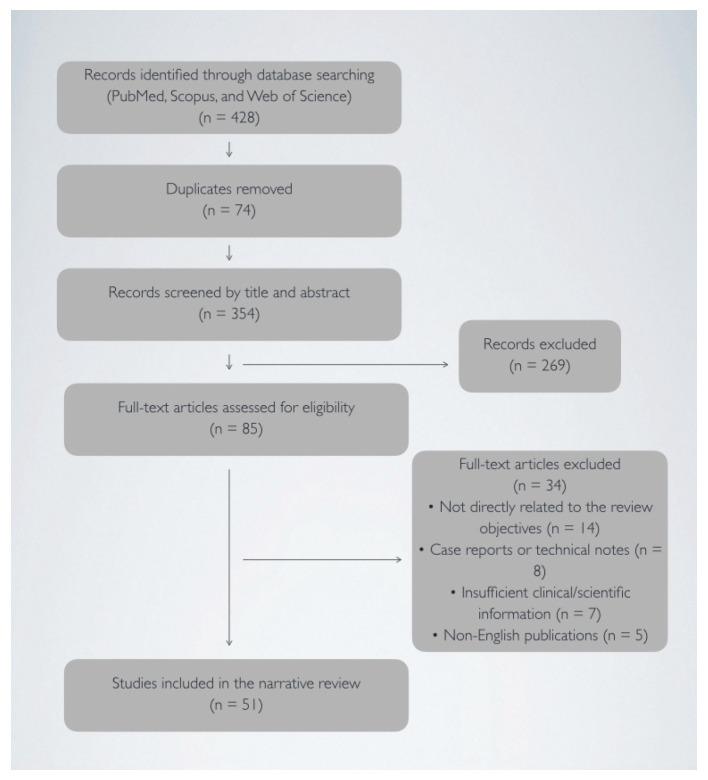
Simplified PRISMA-style flow diagram of the literature selection.

An extensive literature search was conducted using electronic databases, including Scopus, Web of Science, and PubMed, from the last 25 years (2000–2026). Keywords such as “crown lengthening” OR “aesthetic crown lengthening”, “open flap crown lengthening” OR “flapless crown lengthening”, “periodontal surgery” OR “laser-asisted” were used. The literature search identified 428 records from the selected databases. After removal of duplicates, 354 records underwent title and abstract screening. The inclusion criteria were clinical trials, prospective studies, retrospective studies, systematic reviews, case series. The exclusion criteria were editorials, conference abstracts, studies without clinical outcomes, non-English studies. Three independent reviewers screened titles and abstracts. Priority was given to clinical trials, systematic reviews, and comparative clinical studies. Eighty-five full-text articles were assessed for eligibility, of which 51 studies met the inclusion criteria and were incorporated into the narrative review. The selection process is illustrated in a simplified PRISMA-style flow diagram (Figure 1).

## 3. Results and Discussion

### 3.1. Biological Basis of Crown Lengthening Procedure

The supracrestal attachment is essential for the correct clinical performance of the crown lengthening procedure. According to the 2017 World Workshop of the European Federation of Periodontology (EFP) and American Academy of Periodontology (AAP), the term “biologic width” has been replaced by “supracrestal tissue attachment” to more accurately describe the epithelial and connective tissue attachment above the alveolar bone crest.

Previously referred to as the biological width, this attachment is composed of junctional epithelium and connective tissue and plays a vital role in periodontal health and stability [14]. Histological studies have established that the biological width is 2.04 mm, including 0.97 mm for epithelial attachment and 1.07 mm for junctional attachment. However, anatomical variations between 1 and 4 mm have also been reported in the literature [14].

Preservation of the supracrestal attachment after a crown lengthening procedure is crucial. Any damage to this structure can lead to serious complications such as periodontal inflammation, periodontal pocket formation, or gingival recession [15].

### 3.2. Clinical Context of Crown Lengthening

The main indications for crown lengthening include subgingival caries/fractures, impaired passive eruption, and esthetic corrections, with additional restorative needs such as the creation of a ferrule. Deep caries or crown/root fractures extending below the gingiva prevent adequate restorative margins, risking biospace disruption and periodontal pathology. Crown lengthening with flap (ACL) allows access for extraction and restoration placement while establishing a healthy ferrule effect [16].

Impaired passive eruption is a condition characterized by excessive gingival exposure due to incomplete tooth eruption relative to the alveolar bone, classified as type 1 (adequate keratinized tissue) or type 2 (insufficient), subtypes A/B. ACL with gingivectomy or apical repositioned flap corrects clinical short crowns and prevents recurrence [16,17]. Indicated in cases of a gummy smile, irregular gingival contours, or disproportionate tooth-to-gingival ratios, clinical crown lengthening (CCA) harmonizes the smile by apical repositioning of the gums and is often associated with orthodontic treatment or veneering for optimal esthetics [16,17].

Other indications include improving the retention of prosthetic ferrules, repairing perforations, repositioning interprosthetic margins, and compensating for loss of coronal height [16,17].

### 3.3. Crown Lengthening with Flap

The apically positioned flap (APF) technique with bone resection is the primary surgical technique for clinical crown lengthening when soft tissue and bone removal is required to expose more tooth structure. The procedure begins with intrasulcular or submarginal incisions and the elevation of a full-thickness mucoperiosteal flap, usually from the buccal side, and sometimes from the palatal/lingual side. The flap is then elevated apically to allow access and repositioning. Osteotomy and osteoplasty are performed to restore adequate supracrestal tissue attachment and to provide sufficient tooth structure for the margins of the restoration. The amount of bone removed is determined by the desired final position of the gingival margin, usually 3 mm from the restoration margin to the alveolar crest, to allow for stable soft tissue healing and restoration of supracrestal tissue attachment [18,19].

The osteotomy procedure follows the following steps: vertical scoring, root fusion, interproximal bone flattening, and gradual marginal bone augmentation, which can be performed manually or with the aid of rotary instruments [10].

The flap is then repositioned apically and sutured, exposing more of the clinical crown.

Immediate postoperative increase in coronal length is significant, but coronal soft tissue rebound occurs during healing, especially if the flap is sutured close to the bony crest or in cases of thick periodontal biotype. Most tissue repair occurs within the first 3 to 6 months, and the final position of the gingival margin stabilizes within 6 months. The stability of this margin is influenced by the distance between the flap and the osseous crest during closure; a distance of ≥3 mm minimizes rebound and optimizes long-term outcomes [20,21,22].

APF with bone resection is highly predictive of functional and esthetic coronal lengthening, but careful case selection and surgical planning are essential to avoid attachment loss [23].

### 3.4. Flapless Crown Lengthening

Flapless crown lengthening, often performed by piezo surgery or laser, allows for gingival and bone remodeling without a full-thickness flap lift.

When performed by piezo surgery or an erbium laser (e.g., Er:YAG or Er,Cr:YSGG), flapless crown lengthening relies on precise excision and coagulation of soft and hard tissues through the sulcus or through a small crevicular approach, thereby preserving the underlying periosteum and vasculature [4,24,25].

#### 3.4.1. Indications and Prerequisites

Flapless crown lengthening is indicated in cases of limited gingival overhang (impaired passive eruption) and when ≤2–3 mm of bone must be removed to restore a supracrestal tissue attachment of approximately 3 mm and achieve adequate crown height for the planned restoration [4]. Sufficiently attached gingiva and the absence of deep periodontal defects are required. This technique is particularly reliable in the anterior maxilla and in cases of minor bone remodeling [4,12,24].

Clinical and radiographic marginal bone levels, the existing gingival contour, and the future restoration margin are assessed preoperatively to calculate the amount of gingiva and bone that must be removed [4,12]. A periodontal probe is used to mark the desired gingival margin and a 3 mm biologic width zone apical to the future restoration, serving as a reference for soft tissue remodeling and for the depth of laser or piezoelectric osteotomy [4,12].

#### 3.4.2. Laser-Assisted Crown Lengthening

The use of lasers to ablate soft tissue and/or bone is often flapless or minimally invasive, with the potential to reduce morbidity and accelerate healing [26].

Lasers allow the precise cutting of soft and hard tissue, but the practitioner must be fully trained in the specific properties of laser light and its interaction with tissue. For example, diode and Nd:YAG lasers are strongly absorbed by hemoglobin and melanin and are primarily used for cutting soft tissue, with great care needed around teeth due to the risk of damage. In comparison, erbium lasers, such as Er:YAG and Er;Cr:YSGG lasers, are absorbed by water molecules and can be safely used, not only for soft tissue surgery but also for bone surgery. Selective ablation and laser light collimation allow for excellent tissue ablation limited to bone or soft tissue, with the assurance of safe ablation of the bone margin without opening a mucoperiosteal flap [27]. Laser-assisted crown lengthening is indicated for the esthetic management of excessive gingival exposure (e.g., impaired passive eruption), carious exposure, or subgingival fractures, and in cases requiring minimally invasive access to soft and hard tissues. It is particularly suitable for patients seeking reduced morbidity and faster recovery in the anterior esthetic zone [5,20,26,28,29,30].

Flapless techniques are available, particularly with Er:YAG and diode lasers, allowing for the removal of soft tissue and bone without full-thickness flap elevation or suturing [31]. Digital guidance may improve the accuracy and predictability of margin positioning [32].

Laser energy is delivered in controlled pulses for tissue ablation, with minimal collateral damage due to selective absorption and shallow penetration depth [30]. Clinical outcomes include predictable increases in clinical crown length, rapid stabilization of gingival margins (often within 1 month), and minimal tissue resorption (usually <0.3 mm at 9 months) [5,28,29]. Periodontal parameters such as probing depth and attachment remain stable between 6 and 12 months. Patient-reported outcomes have shown high satisfaction, reduced pain, and bleeding compared with conventional flap techniques [28,29,30].

During Er:YAG laser-assisted procedures, bone is typically reduced approximately 3 mm apical to the planned gingival margin to maintain the biologic width [24]. Under local anesthesia, the sulcular epithelium and excess gingiva are laser-removed using a closed-flap technique using soft tissue-friendly settings (e.g., low-energy, high-frequency Er:YAG settings with water spray) to achieve hemostasis and precise contouring without flap elevation [24]. The laser tip is then advanced into the sulcus to perform a controlled osteotomy, removing thin slices of cortical bone following a pre-marked reference line; this avoids significant bone exposure and minimizes periosteal trauma [4,24]. Studies have reported that flapless crown lengthening with an Er:YAG or Er,Cr:YSGG laser allows for significant increases in clinical crown length during the procedure, with stable gingival margins and no need for sutures 3 months postoperatively [24,25].

Compared with conventional methods, this technique has several advantages: reduced postoperative pain and swelling, reduced bleeding, faster healing, and increased patient comfort. The minimally invasive nature of laser surgery preserves healthy tissue and promotes faster recovery, with comparable long-term esthetic and functional outcomes. Laser-assisted crown lengthening is a safe and effective alternative to traditional approaches, especially in esthetically demanding cases [33].

#### 3.4.3. Piezoelectric Crown Lengthening

Piezoelectric surgery allows for flapless osteotomy using ultrasonic inserts calibrated to the bone that cut through mineralized tissue while largely sparing soft tissue and periosteum [12]. After gingivectomy performed with a scalpel or laser, a piezosurgical tip is inserted into the sulcus and used to perform controlled vertical osteotomies, removing the desired amount of bone while preserving the overlying periosteum [12]. This approach has been described in cases of plateless minimally invasive clinical crown lengthening aimed at correcting gingival excess and obtaining a more harmonious and esthetic result [12].

The advantages of the closed-flap crown lengthening procedure are a significant reduction in postoperative pain and swelling, as well as improved early stability of the gingival margin compared with open-flap approaches, as shown in randomized controlled trials [4,34]. Laser-assisted flapless crown lengthening further minimizes tissue trauma and may accelerate clinical outcomes, especially in the anterior esthetic zone [35]. The application of microsurgical techniques using magnification and precision instruments for flapless crown lengthening allows the practitioner to limit tissue destruction and bone resection, with the aim of precisely managing the supracrestal attachment. These methods have demonstrated healthy periodontal conditions and stable clinical attachment levels at one year [6,36].

Minimally invasive crown lengthening techniques are designed to reduce surgical trauma, morbidity, and healing time, while allowing for precise gingival and bone remodeling. The approach should be applied in combination with magnification of the operative field and precise and delicate instruments to minimize tissue trauma and bone resection, with the goal of conservative treatment [6]. However, flapless surgery has some limitations, such as reduced bone visualization and risks of complications or unsightly results.

### 3.5. Digitally Guided Crown Lengthening

Digital guidance and dual guidance techniques, in the context of minimally invasive crown lengthening, refer to the use of advanced digital planning and customized 3D-printed surgical guides to accurately transfer the planned gingival and bone resection positions to the surgical field. These techniques integrate intraoral scans, cone beam computed tomography (CBCT), and digital smile design to create guides that indicate the exact location of the soft tissue incision and bone resection, thereby optimizing esthetic and functional outcomes [32,37,38,39,40,41].

The dual-guide technique uses two separate guides: one for gingivectomy (soft tissue resection) and the other for alveolectomy (bone resection), allowing for very precise execution of both steps. By applying it, a clinician can obtain stable crown length and high esthetic satisfaction at 12 months, comparable to conventional methods [32].

Minimally invasive suturing techniques also contribute to improved wound stability and esthetic outcomes, with high patient satisfaction and papilla preservation over 24 months [42].

Overall, these minimally invasive approaches offer predictable results, reduced morbidity, and favorable esthetic and periodontal outcomes, especially in cases of altered passive eruption and anterior esthetic demands [43,44].

### 3.6. Healing After CLP

The typical healing process after clinical crown lengthening involves an initial phase of soft tissue healing, followed by progressive maturation and stabilization of the gingival margin and restoration of the supracrestal attachment. Immediately following the procedure, there is a marked apical shift in the gingival margin and lengthening of the clinical crown. During the first 3 to 6 months, the gingival margin tends to retract coronally, with most of the tissue repair occurring within the first 3 months, and stabilization is usually achieved within 6 months [2,18,19,20,21,45].

The extent of tissue retraction is influenced by factors such as the position of the flap relative to the alveolar crest at the time of suturing, the periodontal biotype, and the distance between the gingival margin and the bony crest. Thicker biotypes and positioning of the flap closer to the crest are associated with greater coronal retraction [22,46]. Patients may experience minimal discomfort during treatment. It is important to maintain strict oral hygiene and regular professional cleaning. Definitive restorations should be delayed until the gingival margin has stabilized, i.e., at least 3 months for posterior teeth and up to 6 months for anterior teeth, where esthetics are crucial [1,7].

In summary, soft tissue healing and coronal restoration of the gingival margin occur predominantly during the first 3 to 6 months, with final stabilization of the gingival margin and attachment of the supracrestal tissue achieved after 6 months.

### 3.7. Typical Postoperative Complications After Crown Lengthening Procedure

Flap and flapless (minimally invasive) crown lengthening techniques have similar clinical outcomes but differ in terms of postoperative morbidity, occurrence of typical complications, and early healing phase. Flapless techniques are generally associated with less discomfort and faster soft tissue healing, at the cost of a slightly higher risk of gingival recession and more limited access for bone remodeling [9,25,47].

The most common complications after CLP are shown in the Table 1.

Other postoperative complications of the CLP technique reported in the literature include:

- Dentin hypersensitivity, the most common complication (5 to 6% of cases) [50].
- Gingival rebound, characterized by coronal migration of the gingival margin during healing, which may reduce the exposed tooth surface obtained during the procedure [20,21,45].
- Gingival recession, especially in patients with thinned periodontium, which may affect the esthetic outcome.
- Infection or delayed healing, although rare with appropriate technique and adequate postoperative care [51].
- Tooth mobility or migration, especially in case of loss of occlusal adhesion during healing [51].
- Changes in probing depth and clinical attachment level at the treated sites, which may persist for several months after the intervention [52].
- Transient bacteremia, a risk inherent to periodontal surgery [51].

Patient factors, such as smoking and diabetes, are associated with an increased risk of complications. Most complications are mild and do not interfere with favorable surgical outcomes or normal daily activities [50].

### 3.8. Comparison of Open vs. Closed Techniques

Open crown lengthening involves flap elevation with direct visualization of the alveolar bone, allowing precise osseous recontouring and more predictable management of biologic width, whereas closed crown lengthening is performed without flap reflection, using minimally invasive techniques that reduce surgical trauma. Closed procedures are generally associated with faster healing, less postoperative discomfort, and earlier soft tissue maturation, while open techniques require a longer healing period, particularly when esthetic rehabilitation is planned [16,25,49]. From an esthetic perspective, closed crown lengthening may better preserve gingival architecture in carefully selected cases, whereas open crown lengthening provides superior predictability in situations requiring significant bone recontouring or extensive crown exposure [22,53]. Detailed comparison between both procedures is presented in Table 2.

### 3.9. Clinical Decision-Making

Clinical decision-making depends on various factors such as patient factors (biotype, expectations), dental factors, and operator experience.

Periodontal phenotype plays a significant role in both treatment planning and postoperative outcomes following crown lengthening procedures. Patients with a thin periodontal phenotype are generally more susceptible to gingival recession, marginal instability, and the development of interdental “black triangles” due to the reduced thickness of the soft tissues and their limited resistance to surgical trauma. Consequently, even minor alterations in gingival position may have a pronounced esthetic impact, particularly in the anterior region. In contrast, patients with a thick periodontal phenotype tend to demonstrate greater soft tissue resilience and a lower risk of recession; however, they are more prone to coronal rebound of the gingival margin during healing. This rebound phenomenon may delay stabilization of the final gingival architecture and potentially compromise the initially achieved crown length. Therefore, assessment of the periodontal phenotype should be considered an essential component of preoperative evaluation, as it may influence both the selection of the surgical approach and the timing of definitive restorative treatment [2,9,11,16,34].

Patient expectations and anxiety are also important patient-related factors that may influence the choice of procedure. Flapless crown lengthening is associated with less postoperative pain, less swelling, and shorter operative time, resulting in greater patient satisfaction and reduced anxiety, especially in the esthetic zone, and patients who desire “suture-free, minimal downtime” procedures are more likely to be satisfied with flapless techniques [5,9].

Dental factors, such as the need for tooth exposure and anatomical and prognostic factors, are quite important in the clinical decision-making process. Dental indications include altered passive eruption, subgingival caries, irreparable fractures/problems with crown-to-root ratio, and inadequate ferrule; flapless techniques are the best option for cases that require minimal bone resection (≤2–3 mm) and direct gingival shaping [2,16].

Crown-to-root ratio, presence of periodontal defects, width of keratinized tissue, and proximity to frenulum are potential risk factors for long-term recession and postoperative instability, and while conventional flap surgery may still be preferred in cases with complex anatomy or when extensive bone surgery is required, studies have highlighted that adherence to a 3 mm biologic width and adequate ferrule reach (1.5–2 mm) are crucial for outcomes with both techniques [2,16]. Operator experience is of paramount importance for clinical outcomes after CLP. Crown lengthening without a flap is more operator-dependent, as errors in energy settings, ablation depth, or angular control may result in the removal of undercut bone, inadequate clinical exposure of the crown, or inadvertent damage to nearby structures [2,35,55]. Open-flap surgery is more forgiving in terms of visual access and direct control, but problems such as repositioning and tension can cause recession and instability of the gingival margin [2,9,55]. Surgeons experienced with erbium lasers or piezo surgery and with a systematic preoperative planning protocol (CBCT, guided planning with silicone stent where applicable) have reported better predictability of gingival margin stability and reduced rebound with flapless procedures [2,5,11,34,55]. Consultation with a restorative dentist to determine the exact prosthetic margin and degree of tooth exposure also improves outcomes, regardless of the technique chosen [2,11,34].

### 3.10. Limitations of the Current Evidence

- Limited comparative studies and small sample sizes: Most studies of flapless crown lengthening are uncontrolled case series, case reports, or have small sample sizes, which limits the reliability of the evidence-based dentistry (EBD) arguments and the generalizability of the results [2,5,20]. Comparisons between open-flap and flapless techniques often include ≤20–30 patients in the comparison groups, which increases the risk of bias and limits subgroup analyses [2,5,20].
- Variability of surgical techniques and instruments/lack of standardized protocols: Different laser wavelengths (Er:YAG, Er,Cr:YSGG), different pulse settings, different piezoelectric inserts, and different surgical sequences (e.g., flapless vs. minimally invasive flap) are used in studies, making it difficult to compare the two techniques. Furthermore, there is no universally accepted protocol regarding the depth of bone resection, extent of gingivectomy, or time to final restoration [2,5,11,16,34].
- Short follow-up periods: Many studies report results for only 3 to 6 months, while gingival healing and bone remodeling can often continue beyond this period, especially in the esthetic zone. Long-term follow-up data (≥12 months) are limited, so the actual stability of flapless crown lengthening and its long-term impact on periodontal health remain incompletely documented [2,9,34,35].

This narrative review has several methodological limitations that should be acknowledged. First, the review did not follow a fully systematic search protocol, and therefore the possibility exists that some relevant studies may not have been identified, which may affect the comprehensiveness of the evidence synthesis. Second, as an inherent limitation of the narrative review design, the selection of studies is subject to potential selection bias, as inclusion was based on relevance to the predefined objectives rather than on strictly reproducible eligibility criteria.

In addition, no formal quality assessment or risk of bias evaluation of the included studies was performed. As a result, the strength and reliability of individual findings could not be systematically weighted, and conclusions should therefore be interpreted with caution. Finally, the heterogeneity of study designs, clinical protocols, and outcome measures across the included literature limits direct comparability between studies and precludes quantitative synthesis.

## 4. Conclusions

The available literature suggests that flap and flapless crown lengthening techniques should be viewed as complementary approaches rather than competing alternatives, with their selection largely determined by case-specific anatomical and clinical considerations. Flap-based techniques may provide improved access and visibility of the underlying osseous structures, which can facilitate more controlled bone recontouring in complex cases or situations requiring extensive surgical modification.

In contrast, flapless approaches appear to be associated with potential advantages in selected cases, including reduced surgical trauma, shorter operative time, and improved early postoperative comfort. However, these potential benefits should be interpreted with caution, as evidence is primarily derived from heterogeneous studies with variable designs, follow-up periods, and outcome measures.

Case selection remains a critical determinant of treatment success, with factors such as gingival biotype, keratinized tissue availability, and the extent of required bone reduction influencing the choice of technique. While some studies suggest comparable short-term outcomes between approaches in well-selected cases, limitations in study quality and heterogeneity prevent definitive comparative conclusions.

Periodontal phenotype should be considered a key determinant of treatment outcomes, as thin phenotypes may be associated with a greater risk of recession and marginal instability, whereas thick phenotypes may exhibit increased gingival rebound and delayed tissue stabilization.

Overall, due to the narrative nature of this review and the variability of the underlying evidence, no technique can be considered universally superior. Instead, optimal outcomes depend on individualized treatment planning based on biological principles, anatomical conditions, and clinician expertise. Further well-designed prospective studies with standardized outcome measures and long-term follow-up are needed to strengthen the evidence base and improve comparability between techniques.

The current evidence supporting flapless crown lengthening remains limited by heterogeneity, relatively small sample sizes, and variable follow-up periods; therefore, further high-quality randomized controlled trials are required before definitive clinical recommendations can be established.

## Figures and Tables

**Table 1:** Most common complications after crown lengthening procedures.

Technique	Complication/Outcome	Explanation	Source
Flap technique	Bleeding and need for sutures	Reflecting a full-thickness flap often causes more intraoperative bleeding and usually requires sutures. Sutures may cause discomfort, plaque retention, or wound dehiscence.	Dayoub & Yousef, 2019 [48]; Ribeiro et al., 2014 [9]
	Swelling and postoperative pain	Flap elevation and periosteal stripping create greater surgical trauma, commonly leading to more edema and higher pain levels during the first postoperative days compared with flapless methods.	Oqlah et al., 2025 [47]; Tianmitrapap et al., 2022 [25]
	Wound dehiscence and delayed healing	Larger surgical wounds and tension around sutures may increase the chance of wound opening or slower healing, especially with poor plaque control or local irritation.	Dayoub & Yousef, 2019 [48]; Ribeiro et al., 2014 [9]
Flapless technique	Gingival rebound	Coronal regrowth or rebound of the gingival margin may occur over time. Some studies suggest slightly greater rebound in flapless cases, particularly in thick gingival biotypes.	Crosby et al., 2023 [11]; Altayeb et al., 2022 [49]; Oqlah et al., 2025 [47]
	Technical limitations/operator dependency	Limited direct visualization can make osteotomy less precise. Inexperienced handling may lead to insufficient bone removal or accidental damage to nearby tissues.	Oqlah et al., 2025 [47]; Tianmitrapap et al., 2022 [25]
	Less bleeding and fewer suture-related issues	Because no flap is reflected, flapless procedures generally produce less bleeding and usually avoid sutures, reducing suture discomfort and related complications.	Tianmitrapap et al., 2022 [25]; Dayoub & Yousef, 2019 [48]

**Table 2:** Comparison of open vs closed crown lengthening procedures.

Feature	Open Crown Lengthening	Closed Crown Lengthening
Definition	Surgical procedure involving flap elevation to expose bone and root structure	Minimally invasive procedure without raising a mucoperiosteal flap
Technique	Gingival tissue is incised and reflected (flap opened), bone may be reshaped	Tissue is removed or reshaped externally, often with lasers or electrosurgery. The bone is remodeled through the sulcular incision without raising a flap.
Visibility for surgeon	Direct visualization of bone and root	Limited visibility (performed “blind”)
Bone removal (osteotomy)	Yes	Yes
Healing time	Longer (2–6 weeks initial healing; full healing may take months) [16,49]	Faster healing (1–2 weeks typical) [25,49]
Postoperative discomfort	Higher short-term pain, swelling, anxiety [9,25]	Lower pain, less bleeding, better comfort and satisfaction [25,53]
Sutures required	Yes	Usually not required
Bleeding	More intraoperative bleeding	Less bleeding, especially with laser use
Esthetic outcomes	Good, but higher risk of marginal irregularities if flap tension exists [22]	Very satisfactory, smoother contour and less tissue loss [53,54]
Gingival rebound	Mean rebound ~0.2–0.3 mm, higher in thick biotypes [5,22]	Comparable rebound but often more predictable in thin biotypes [5,25]
Periodontal stability	Stable, PPD reduction, no significant difference vs. flapless [9,25]	Similarly stable periodontal parameters [9,25]
Risk of complications	Slightly higher (infection, flap issues, bone exposure)	Lower overall risk
Equipment needed	Standard surgical instruments	May involve laser or electrosurgery devices
Procedure time	Longer	Shorter

## Data Availability

No new data were created or analyzed in this study.
